# Graphene-Based Biosensors for Detection of Composite Vibrational Fingerprints in the Mid-Infrared Region

**DOI:** 10.3390/nano9101496

**Published:** 2019-10-20

**Authors:** Yijun Cai, Yanfen Hang, Yuanguo Zhou, Jinfeng Zhu, Jingwen Yang, Xuanyu Wang

**Affiliations:** 1 Fujian Provincial Key Laboratory of Optoelectronic Technology and Devices, Xiamen University of Technology, Xiamen 361024, China; yijuncai@foxmail.com (Y.C.); wxyx815@163.com (X.W.); 2 College of Communication and Information Engineering, Xi’an University of Science and Technology, Xi’an 710054, China; yanfenhang@foxmail.com; 3 Department of Electronic Science, Xiamen University, Xiamen 361005, China; nanoantenna@hotmail.com (J.Z.); 35320172200252@stu.xmu.edu.cn (J.Y.)

**Keywords:** label-free biosensors, metasurface, graphene, surface plasmons, nanophotonics

## Abstract

In this study, a label-free multi-resonant graphene-based biosensor with periodic graphene nanoribbons is proposed for detection of composite vibrational fingerprints in the mid-infrared range. The multiple vibrational signals of biomolecules are simultaneously enhanced and detected by different resonances in the transmission spectrum. Each of the transmission dips can be independently tuned by altering the gating voltage applied on the corresponding graphene nanoribbon. Geometric parameters are investigated and optimized to obtain excellent sensing performance. Limit of detection is also evaluated in an approximation way. Besides, the biosensor can operate in a wide range of incident angles. Electric field intensity distributions are depicted to reveal the physical insight. Moreover, another biosensor based on periodic graphene nanodisks is further proposed, whose performance is insensitive to the polarization of incidence. Our research may have a potential for designing graphene-based biosensor used in many promising bioanalytical and pharmaceutical applications.

## 1. Introduction

Surface plasmon resonance (SPR) biosensors have received tremendous attention over the past decades due to their label-free sensing ability [1,2]. They utilize surface plasmon polariton (SPP) waves to detect the refractive index (RI) change in the sensing surface produced by the alteration of biomolecule concentration. A variation in the propagation constant of SPP caused by the change of RI can be optically measured for inverse calculation of biomolecular RI. Nevertheless, in order to identify the species of biomolecule, roughly probing the change of RI in the biological processes is not sufficient. Characterization of biomolecules through their vibrational properties is also necessary to differentiate constituent biomolecular species. Vibrational fingerprints relating with the valuable molecular information are mainly located at the mid-infrared range, which uniquely identify the biomolecules in a multitude of biological processes. Infrared spectroscopy is widely used as a nondestructive label-free technique to access these vibrational fingerprints and provide exquisite biochemical information in bulk materials. However, vibrational signals in minute amounts of analytes are quite weak due to the mismatch between nanometric size of biomolecules (<10 nm) and the mid-infrared wavelengths (2 to 6 μm). To solve this problem, resonant metallic nanoantennas are commonly used to enhance light-matter interaction due to highly concentrated and enhanced infrared near fields [3,4,5,6,7], which is also called surface-enhanced infrared absorption (SEIRA). Although the resonant frequency can be tuned by adjusting the geometrical size of nanoantennas, biomolecules with spectrally separated vibrational bands usually require dual- or multiband plasmonic structures to minimize the number of false positive results [8,9]. On the other hand, to distinguish multiple analytes in heterogeneous biological samples, several multi-resonant infrared metallic or dielectric metasurfaces have been developed to simultaneously detecting the composite vibrational signals of different biomolecules [10,11,12].

Although the multi-resonant biosensors based on traditional materials can probe biomolecules in a relatively wide frequency range, their detected bands cannot be dynamically tuned to the designing frequencies after the fabrication process. To realize the postfabrication tuning, graphene has been taken into account as a promising candidate for biosensors. Graphene is a two-dimensional material with carbon atoms arranged in a honeycomb lattice [13,14]. Graphene-based photonic devices have attracted great interest due to its exceptional electrical and optical properties [15,16,17,18]. Optical biosensors based on graphene possess the advantage of tunable spectral selectivity owing to the tunability of graphene conductivity by electrostatic gating. Besides, the strong spatial light confinement in graphene also contributes to an enhanced sensitivity in the detection of RI changes and vibrational signals. In 2004, researchers from IBM Corporation utilized graphene nanoribbons to detect surface-adsorbed thin films of polymer for the first time [19]. They further investigated the coupling between graphene plasmons and the vibrations of solid- and gas-phase molecules [20]. Rodrigo et al. proposed a plasmonic graphene-based biosensor to identify the vibrational fingerprints of protein [21]. They experimentally demonstrated that dynamic resonance tuning could be achieved by varying the gating voltage and vibrational signals were significantly enhanced compared with gold nanoantennas. Hu et al. successively reported thin polymers sensing and gas identification with graphene plasmon, utilizing CaF_2_ nanofilm instead of SiO_2_ to avoid the plasmon-phonon hybridization [22,23]. Zhu et al. designed a hybrid metasurface with suspended graphene and gold nanoantennas, while the nanoantennas were deposited close enough (about 10 nm), ultrasensitive biosensing ability was achieved to probe low-molecular-weight analytes [24]. The above reported graphene-based biosensors showed higher sensitivity and detection limit than traditional biosensors. However, they all suffered from the problem of single resonant property, which limited their applications in detecting composite vibrational fingerprints simultaneously.

In this paper, a label-free multi-resonant graphene-based biosensor (MRGB) is numerically proposed for detection of composite vibrational fingerprints in the mid-infrared region. Compared with the previous structures [18,19,20,21,22,23,24], the proposed biosensor requires simpler fabrication techniques. Each of the plasmonic resonances in transmission spectrum can be independently tuned by gating voltages to overlap with the vibrational fingerprints of different biomolecules at designing frequencies, leading to a wide spectral detection range. The influences of geometric parameters and incident angles on the performance of MRGB are discussed. Moreover, MRGB with graphene nanodisks are further designed to guarantee superior sensing ability under incident light with different polarizations.

## 2. Modeling and Methods

**Figure 1:**
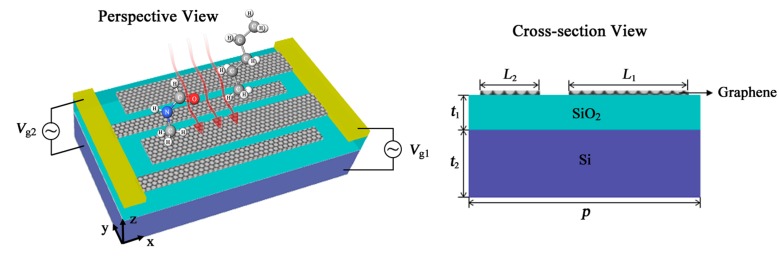
Schematic of multi-resonant graphene-based biosensor (MRGB). *L*_1_ and *L*_2_ (*L*_1_ > *L*_2_) denote the widths of the wider and narrower graphene nanoribbons, respectively. *t*_1_ and *t*_2_ are the thicknesses of SiO_2_ and Si. *p* is the periodic graphene nanoribbons (GNRs) array pitch.

In order to realize a biosensor for detection of composite vibrational fingerprints, we utilize periodic graphene nanoribbons (GNRs) as a multi-resonant metasurface as plotted in Figure 1. GNRs can be grown by chemical vapor deposition and wet-transferred to the surface of SiO_2_ dielectric layer, while Si is chosen as the substrate to support this device. GNRs with different widths can be exposed using electron beam lithography. Wider GNRs are connected to the metallic electrode at the right far ends as the top contact, while narrower GNRs are connected to the metallic electrode at the left far ends as another top contact. The doped Si substrate is used as the bottom contact. Two bias voltages, *V_g_*_1_ and *V_g_*_2_, are applied on the interdigitated GNRs to control the Fermi energies *E_F_*_1_ and *E_F_*_2_ of GNRs separately. We assume that a mid-infrared plane wave illuminates on the top surface of MRGB, interacting with the graphene metasurface and the adsorbed biomolecules. The interaction is investigated using COMSOL Multiphysics, which solves Maxwell Equations with finite element method (FEM) in frequency domain. Floquet periodicity is chosen as boundary condition in both *x*- and *y*-directions. Tetrahedral meshes are used in the entire domain. The transmittance of MRGB can be expressed as *T* = |S_21_|^2^, where S_21_ can be directly obtained from the user-defined ports in the *z*-directions.

The permittivity of SiO_2_ and Si used in the simulation are chosen from Ref. [25]. In order to reduce the mesh number and improve the computational efficiency in simulation, we assume graphene as a 2D conductive surface without thickness, instead of a 3D bulk volume. This assumption has already been proved to be effective and efficient in the simulation of graphene-based devices before [26]. The surface conductivity of graphene can be calculated according to the well-known Kubo formulas [27]:

(1) $$\sigma (\omega ,E_{F},\Gamma ,T)=\sigma_{\mathrm{int}ra}+\sigma_{\mathrm{int}er}\;$$

(2) $$\sigma_{\mathrm{int}ra}=\frac{je^{2}}{\pi \hbar^{2}(\omega -j2\Gamma )}\int_{0}^{\infty }\xi \left(\frac{\partial f_{d}(\xi ,E_{F},T)}{\partial \xi }-\frac{\partial f_{d}(-\xi ,E_{F},T)}{\partial \xi }\right)d\xi \;$$

(3) $$\sigma_{\mathrm{int}er}=-\frac{je^{2}(\omega -j2\Gamma )}{\pi \hbar^{2}}\int_{0}^{\infty }\frac{f_{d}(-\xi ,E_{F},T)-f_{d}(\xi ,E_{F},T)}{{\left(\omega -j2\Gamma \right)}^{2}-4{\left(\xi /\hbar \right)}^{2}}d\xi$$

(4) $$\;f_{d}\left(\xi ,E_{F},T\right)={\left(e^{(\xi -E_{F})/k_{B}T}+1\right)}^{-1}\;$$

Here, *σ_intra_* and *σ_inter_* are the intraband and interband contributions, respectively. *ω* is the angular frequency, *E_F_* denotes the Fermi energy, *Γ* is the scattering rate with *Γ* = 1/(2*τ*), *τ* is the relaxation time of electron-phonon, *T* is the Kelvin temperature, *ħ* is the reduced Planck constant, *k_B_* is the Boltzmann constant, *e* and *ξ* are the electron charge and energy, *f_d_*(*ξ, E_F_, T*) is the Fermi–Dirac distribution.

In the mid-infrared region, the intraband transition dominates the light-graphene interaction since the interband transition can be ignored. When *T* is assumed to be 300 K, Kubo formulas can be simplified as follows:

(5) $$\sigma_{g}=\frac{ie^{2}E_{F}}{\pi \hbar^{2}(\omega +i\tau^{-1})}\;$$

The realistic value of *τ* for graphene grown on the SiO_2_ substrate is usually smaller than 100 fs. To guarantee the validity and reliability of our simulation model, we choose *τ* as 15 fs according to the experimental work form Ref. [21], so the surface conductivity of graphene is determined by the Fermi energy *E_F_* and angular frequency *ω*. An approximate closed-form expression between *E_F_* and gating voltage *V_bias_*, is given by [28] as Equation (6):

(6) $$E_{F}=\hbar v_{f}\sqrt{\frac{\pi \varepsilon_{0}\varepsilon_{r}V_{bias}}{eD}}$$

where *ε*_0_ is the permittivity of free space, *ε_r_* and *D* are the equivalent relative permittivity and the thickness of insulating layers, respectively.

To demonstrate the detection ability for composite vibrational fingerprints of multiple biomolecules, we assume the protein and lipid molecules in the simulation as a thin layer with 8 nm thickness according to the previous experimental work [21], whose relative permittivity can be calculated by the Lorentzian series as:

(7) $$\varepsilon_{m}(\omega )=n_{\infty }^{2}+\sum_{k=1}^{N}\frac{S_{k}^{2}}{\omega_{k}^{2}-\omega^{2}-i\omega \gamma_{k}}$$

The symbol *N* denotes the number of oscillators, which is assumed as 3 relating to the corresponding parameters. The main vibrational fingerprints of proteins are amide I and II bands (about 1668 cm^−1^ and 1532 cm^−1^), while the CH_2_ band (about 2900 cm^−1^) is dominated in lipid molecules. Therefore, parameters are chosen as *ω*_1_ = 1668 cm^−1^, *ω*_2_ = 1532 cm^−1^, *ω*_3_ = 2900 cm^−1^, *γ*_1_ = 78.1 cm^−1^, *γ*_2_ = 101 cm^−1^, *γ*_3_ = 111 cm^−1^, *S*_1_ = 213 cm^−1^, *S*_2_ = 200 cm^−1^, *S*_3_ = 230 cm^−1^,$n_{\infty }^{2}=2.08$, according to the experiment works from Refs. [11,21,29].

In our simulations, the exact sensitivity of MRGB is difficult to calculate due to the rough approximation of the biomolecules. However, the changing law of its sensitivity can be derived using the enhancement factor (*EF*), which can be expressed as [4]:

(8) $$EF=\frac{I_{SEIRA}}{I_{0}}\times \frac{A_{0}}{A_{SEIRA}}$$

Here, *I*_0_ denotes the unenhanced signal strength, *A*_0_ and *A_SEIRA_* are the areas covered with molecules in reference and SEIRA measurements. *I_SEIRA_* is the enhanced signal strength that can be obtained from the baseline-corrected vibrational signal. For our simulation, variation of any parameter has no effect on *I*_0_, *A*_0_ and *A_SEIRA_*. Therefore, the changing law of sensitivity is directly related to the variation rule of *I_SEIRA_*, which can be approximately observed from the simulated transmittance spectra.

## 3. Results and Discussion

**Figure 2:**
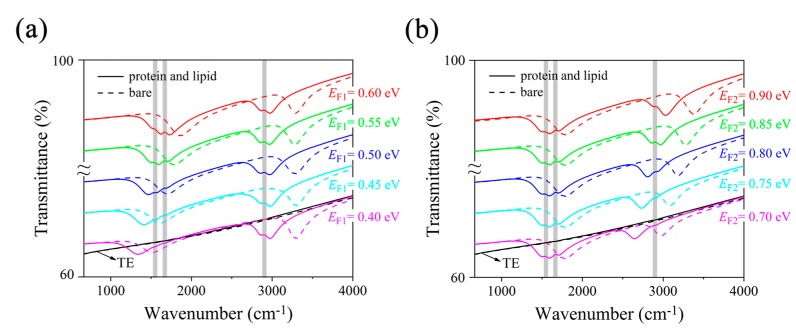
Transmittance spectra of MRGB under transverse electric (TE) (black curves) and transverse magnetic (TM) (colored curves) mid-infrared incidence while sweeping *E_F_*_1_ (**a**) and *E_F_*_2_ (**b**) of graphene, with (solid curves) and without (dashed curves) protein and lipid molecules. Gray vertical bars represent vibrational bands of protein and lipid molecules. Curve with larger *E_F_* is translated upward with 6% in order to demonstrate the spectral shift clearly.

Transmittance spectra of MRGB under mid-infrared incidence are plotted in Figure 2 to investigate the detection capacity for composite vibrational fingerprints of multiple biomolecules. The geometric parameters are *L*_1_ = 40 nm, *L*_2_ = 20 nm, *t*_1_ = 280 nm, *t*_2_ = 500 nm, and *p* = 80 nm. Two obvious transmission dips are observed in the spectra under TMincidence, which is able to induce the electrons to vibrate in the finite width of GNRs due to its electric field direction. In contrast, TE incidence could not excite the plasmonic resonance in GNRs because of its electric field parallel to the nanoribbons. Hence, we focus on the sensing performance of MRGB with TM incidence in the following analysis.

In Figure 2a, by sweeping *E_F_*_1_ from 0.40 eV to 0.60 eV, the first transmission dip of MRGB without protein and lipid is electrostatically tuned from 1525 cm^−1^ to 1875 cm^−1^, while the second resonance frequency is located around 3300 cm^−1^, with *E_F_*_2_ fixed at 0.85 eV. After the adsorption of protein and lipid molecules, the spectra are red-shifted due to the RI change at the surface of MRGB. Although the thickness of biomolecule layer is only several nanometers, the detected frequency shifts still exceed 350 cm^−1^, which is better than Ref. [21]. Besides, two vibrational fingerprints at 1668 cm^−1^ and 1532 cm^−1^ are almost undetectable as the resonance dip is far from them (e.g., for *E_F_*_1_ = 0.40 eV). As the spectral overlap increases, the vibrational signals become progressively more intense (e.g., for *E_F_*_1_ = 0.60 eV), whose spectral positions are in accordance with the amide I and II bands, respectively.

In Figure 2b, the second resonant transmission dip is tuned by gating voltage *V_g_*_2_ to sweep over the lipid vibrational band independently. When *E_F_*_1_ = 0.55 eV and *E_F_*_2_ = 0.70 eV, the vibrational bands in protein are easily detected attributed to the first transmission dip. However, the lipid vibrational band is almost undetectable since it is far from the second transmission dip. As *E_F_*_2_ increases to 0.85 eV, the lipid sensing is achieved by narrow peak appearing at 2900 cm^−1^ corresponding to the CH_2_ band. It unambiguously reveals the presence of the lipid compounds in a chemically specific manner. If *E_F_*_2_ continues increasing to 0.90 eV, the detected lipid vibrational signal becomes weakened as the second resonant dip moves away to larger frequencies gradually.

Therefore, the proposed MRGB can not only work as a refractive index biosensor by detecting a plasmon resonance spectra shift, but also identify composite vibrational fingerprints in multiple biomolecules simultaneously due to the resonant coupling between plasmons and molecular vibrations [30]. Moreover, it possesses the independent tuning ability for individual plasmonic resonance, which is significant and flexible in practical sensing applications.

**Figure 3:**
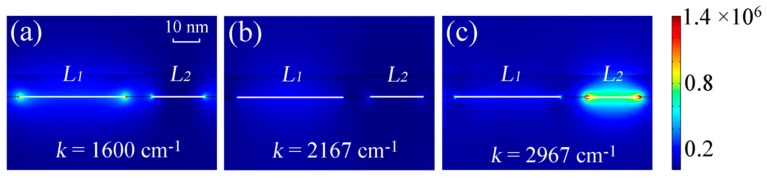
Side view of electric field distributions of MRGB with protein and lipid at some specific wavenumbers for (**a**) *k*=1600 cm^−1^, (**b**) *k*=2167 cm^−1^, (**c**) *k*=2967 cm^−1^ under normal TM incidence, where *L*_1_ = 40 nm, *L*_2_ = 20 nm, *t*_1_ = 280 nm, *t*_2_ = 500 nm, *p* = 80 nm, *E_F_*_1_ = 0.55 eV, *E_F_*_2_ = 0.85 eV. The white lines denote the outline of GNRs.

We depict the electric field distributions in Figure 3 to illustrate the resonant property of MRGB with protein and lipid molecules. As can be seen from Figure 2, when *E_F_*_1_ = 0.55 eV and *E_F_*_2_ = 0.85 eV, the transmission spectra show two obvious dips around 1600 cm^−1^ and 2967 cm^−1^. At *k* = 1600 cm^−1^, the enhanced electric field is dramatically concentrated along the edges of the wider GNRs as shown in Figure 3a. This is attributed to the fact that the incident TM mid-infrared light can excite electrons of graphene to oscillate in the finite width of nanoribbon, inducing the localized surface plasmon resonance (LSPR) in the wider GNRs [31,32]. Similarly, at *k* = 2967 cm^−1^ as shown in Figure 3c, plasmonic hotspots are generated at the edges of the narrower GNRs. The localized resonance originates from the electric dipole surrounding the edges of narrower GNRs and contributes to the corresponding transmission dip in the spectrum. Compared with gold nanoribbons, a higher field confinement for similar infrared-frequency plasmons is observed due to the atomic thickness of graphene as demonstrated in Ref. [21]. Consequently, a much larger spatial overlap between the plasmonic near field and biomolecules occurs, leading to a better sensitivity than biosensor based on metallic nanoantennas [21].

On the other hand, there is no obviously enhanced electric field at *k* = 2167 cm^−1^ as shown in Figure 3b. The explanation is that this frequency is far from either of the two resonance dips in the spectral domain.

**Figure 4:**
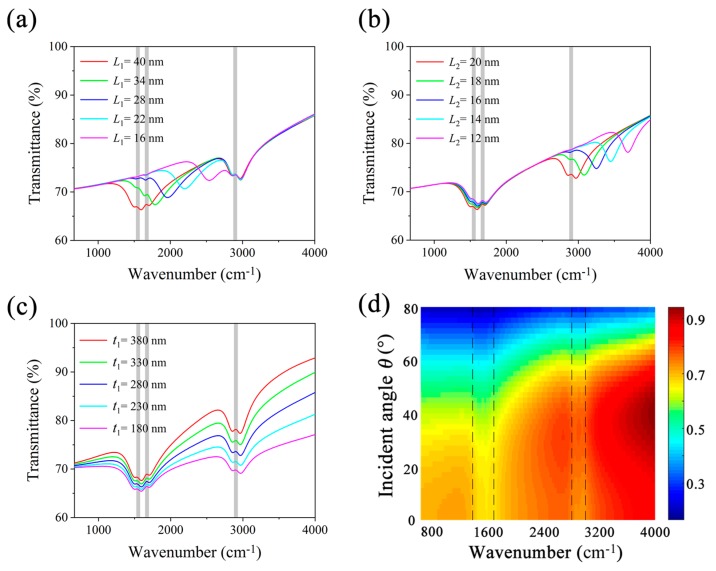
(**a**–**c**) Transmittance of MRGB covered with protein and lipid molecules under normal TM incidence, for *p* = 80 nm, *t*_2_ = 500 nm, *E_F_*_1_ = 0.55 eV, *E_F_*_2_ = 0.85 eV. (**a**) Using different *L*_1_ values, for *L*_2_ = 20 nm, *t*_1_ = 280 nm. (**b**) Using different *L*_2_ values, for *L*_1_ = 40 nm, *t*_1_ = 280 nm. (**c**) Using different *t*_1_ values, for *L*_1_ = 40 nm, *L*_2_ = 20 nm. Gray vertical bars represent vibrational bands of protein and lipid molecules. (**d**) Transmittance as a function of wavenumber and incident angle *θ* under TM incidence, where *L*_1_ = 40 nm, *L*_2_ = 20 nm, *t*_1_ = 280 nm, *t*_2_ = 500 nm, *p* = 80 nm, *E_F_*_1_ = 0.55 eV, *E_F_*_2_ = 0.85 eV. Dashed vertical black strips outline the vibrational bands of protein and lipid molecules.

The widths of GNRs are critical to the plasmonic resonance in MRGB. By gradually adjusting *L*_1_ from 40 nm to 16 nm, the first transmission dip can be tuned from 1600 cm^−1^ to 2550 cm^−1^ independently as shown in Figure 4a. These two transmission dips tend to merge together as *L*_1_ becomes smaller and smaller, then MRGB turns out to be a broadband graphene-based biosensor (BGB), which can be used for covering several vibrational lines within a limited frequency range. Similarly, the second transmission dip can also be tuned independently by changing *L*_2_ as demonstrated in Figure 4b. The resonant frequency has a blue shift as the width of GNRs decreases, which is mainly attributed to the decrease of the effective length for dipole oscillation along the nanoribbon edges. The natural resonant frequencies of graphene nanoribbons determined by the ribbon width are given by Equation (9) [33,34]:

(9) $$\omega_{\mathrm{res}}≅0.62\sqrt{\frac{e^{2}\times E_{F}}{\hbar^{2}\varepsilon_{0}\varepsilon_{r}L}}$$

One can see from Equation (9) that the resonant frequency *ω_res_* scales with the reciprocal of the square root of the width of graphene nanoribbon.

Another geometric parameter playing a vital role in the MRGB performance is the thickness of dielectric layer *t*_1_. Figure 4c depicts that transmission dip will have a dramatic drop with the decrease of *t*_1_. However, the strength of resonance is also lessened, which impedes the sensitivity of MRGB. Thus, there is an optimal thickness *t*_1_, at which the performance of MRGB reaches the optimum. The optimal *t*_1_ is related with the concerning vibration signals. Considering the relatively strong signal strengths for both protein and lipid, *t*_1_ is chosen as 280 nm as the optimal value in our simulation.

The discussion above is about normal incidence, the sensing performance of MRGB under oblique incidence is further revealed to satisfy different sensing applications. Transmittance spectra of MRGB covered with protein and lipid molecules under different incident angles are plotted in Figure 4d. As can be seen, two obvious transmission dips remain at around 1600 cm^−1^ and 2900 cm^−1^ when *θ* < 60°. They overlap with the vibrational fingerprints of corresponding biomolecule in the spectrum. As a consequence, three vertical lines, which indicate the amide-I, amide-II and CH_2_ absorption bands respectively, line in the regions designated by the dashed lines. Thus, sensing performance of MRGB under oblique incidence shows good robustness, which is significant for optical biosensors.

**Figure 5:**
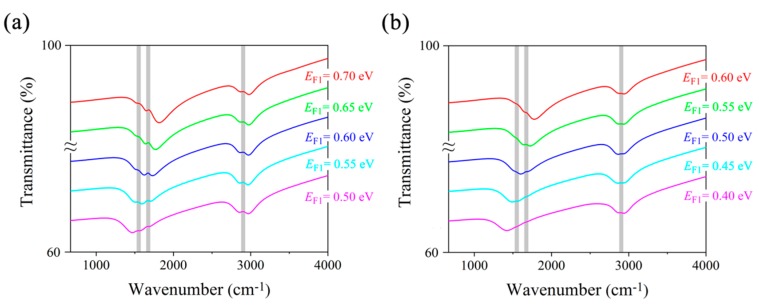
Transmittance spectra of MRGB with different *E_F_*_1_ while accessing different thicknesses of biomolecular layer. (**a**) For biomolecular layer of 8 nm, *E_F_*_2_ = 0.85 eV. (**b**) For biomolecular layer of 2 nm, *E_F_*_2_ = 0.75 eV. Other parameters are the same as in Figure 2. Curve with larger *E_F_* is translated upward with 6% in order to demonstrate the spectral shift clearly.

The detection limit is another critical figure of merit for a biosensor, which represents how little material it can access. In our simulations, we assume the hybridization of protein and lipid as a homogeneous thin layer covering the entire structure including the antennas and substrate. However, the molecules outside the active area contribute little to the enhanced vibrational signals, since SEIRA signals mainly originate from the molecules located in the antenna hot-spots. Therefore, the exact number of molecules required for detection is hardly to be estimated in the simulations. Nevertheless, to investigate the limit of detection roughly, we vary the thickness of biomolecular layer and tune the resonance to match the vibrational frequency as shown in Figure 5. As can be seen, as the thickness of biomolecular layer decreases from 8 nm to 2 nm, the enhanced signal strength gets weakened. However, even when the biomolecular layer becomes as thin as 2 nm, corresponding to essentially a single protein monolayer, the enhanced vibrational signal can also be faintly observed. Therefore, the smallest material that the sensor can access is considered to be monolayer molecules. To obtain *I_SEIRA_* in Equation (8), one should iteratively estimate baseline transmittance *T_baseline_* using a smoothing algorithm [35], and then calculate the baseline-corrected vibrational signal *T_meas_*/*T_baseline_*, where *T_meas_* is the simulated transmittance. The signal strength *I_SEIRA_* of the enhanced molecular vibrational mode is 0.009, which is obtained as peak-to-peak value from the baseline-corrected vibrational signal spectrum. The unenhanced signal strength *I*_0_ is 0.00006, which can also be obtained by the baseline-corrected algorithm. For the proposed MRGB with GNRs, we assume a finite length of GNRs as *l* in a single unit cell along the *x*-axis, so *A*_0_ can be expressed as *A*_0_ ≈ *p* × *l*. The enhanced SEIRA signal mainly originates from the molecules located in the GNRs hot-spots as shown in Figure 3. On the basis of this finding, a commonly used approximation for the active area is the surface of the GNR edges. Therefore, *A_SEIRA_* can be expressed as *A_SEIRA_*≈ 2Δ× *l* (Δ≪*p*), where Δ  is the thickness of GNRs. Therefore, when Δ is assumed as 0.5 nm, by substituting these variables into Equation (8), *EF* can be obtained as 12,000. Obviously, the approximation is rather rough, but it still provides some guidance for experiments.

## 4. MRGB with Graphene Nanodisks

**Figure 6:**
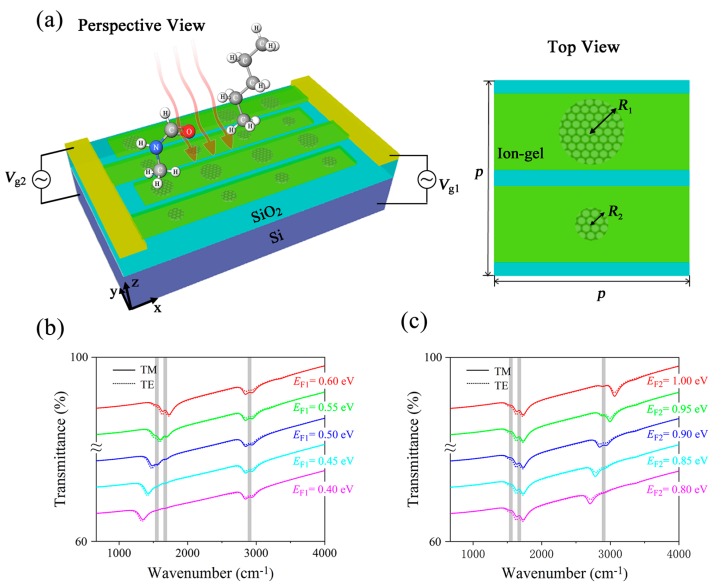
(**a**) Schematic of the MRGB consisting of periodic graphene nanodisks. *R*_1_ and *R*_2_ (*R*_1_ > *R*_2_) are the radiuses of the bigger and smaller graphene nanodisk, respectively. (**b**,**c**) are the transmittance of 3D MRGB covered with protein and lipid molecules while sweeping *E_F_*_1_ (**b**) and *E_F_*_2_ (**c**) of graphene under normal TM (solid curves) and TE (dashed curves) incidence. Gray vertical bars represent vibrational bands of protein and lipid molecules. Curve with larger *E_F_* is translated upward with 6% in order to demonstrate the spectral shift clearly.

GNRs have an infinite length in the *x*-direction compared with the width in the *y*-direction. Thus, the performance of MRGB with GNRs is highly dependent on the polarization of incidence as shown in Figure 2. It can only operate properly under TM polarized incidence, which limits its further application for detection of composite vibrational fingerprints. To overcome the drawback of polarization dependence, we develop an improved three-dimensional (3D) MRGB as illustrated in Figure 6a. A unit of 3D MRGB is made up of two graphene nanodisks (GNDs), SiO_2_ layers and Si substrate. Here, the structural variation of the GNDs in the *x*-direction gives the third dimension compared with GNRs. Two nanodisks have different radiuses denoted as *R*_1_ and *R*_2_, and we assume *R*_1_ > *R*_2_ in the physical model. In the practical applications, the ion-gel layer is commonly considered as a top gate to achieve the tunability of the graphene Fermi energy [36,37]. As plotted in Figure 6a, two sets of interdigitated ion-gel layers with ultra-high capacitance can be spin-coated on top of the proposed MRGB, and two gold gate contacts are fabricated on the interdigitated ion-gel layers to serve as two separated electrodes for the purpose of electrostatic doping. GNDs with different radiuses are controlled by separated top gate voltages, so one can tune Fermi energy of graphene effectively with different bias voltages. The influence on the sensitivity after introducing the ion-gel layer can be neglected due to its ultrathin thickness. The transparent ion-gel can also act as a protective layer to keep GNDs from environmental-induced degradation.

Transmittance spectra of 3D MRGB with protein and lipid under mid-infrared incidence are plotted in Figure 6b,c to investigate the sensing performance for detection of composite vibrational fingerprints in multiple biomolecules. After rigorous optimization for the sensing performance, the geometric parameters are chosen as *R*_1_ = 20 nm, *R*_2_ = 10 nm, *t*_1_ = 280 nm, *t*_2_ = 500 nm, and *p* = 80 nm. One can see two obvious transmission dips in the spectra under both TM and TE incidence. In Figure 6b, when *E_F_*_1_ = 0.40 eV, the resonance dip is far from the amide I and II bands, so the vibrational signals of protein are almost undetectable. By continuously varying *E_F_*_1_ from 0.40 eV to 0.60 eV, the first transmission dip of MRGB is tuned from 1350 cm^−1^ to 1700 cm^−1^, sweeping over the two vibrational fingerprints in protein located at 1668 cm^−1^ and 1532 cm^−1^. The molecular vibrational bands of protein are clearly observed due to the increase of spectral overlap. On the other hand, with *E_F_*_2_ fixed at 0.90 eV, the second resonant dip remains around 2900 cm^−1^, resulting in a detection of CH_2_ band in lipid. In Figure 6c, the first transmission dip is fixed at around 1700 cm^−1^ with *E_F_*_1_ set as 0.60 eV, then the vibrational bands in protein are easily detected attributed to the large spectral overlap. Besides, the second resonance is tuned by bias voltage *V_g_*_2_ to sweep across the lipid vibrational band independently. When *E_F_*_2_ = 0.80 eV, the lipid vibrational signal can hardly be detected since it is far from the second transmission dip. As *E_F_*_2_ increases to 0.90 eV, the detection of lipid molecules is achieved by enhancing a vibrational signal at 2900 cm^−1^ corresponding to the CH_2_ band. If *E_F_*_2_ continues increasing to 1.00 eV, the detected lipid vibrational signal almost vanishes as the second resonant dip moves away gradually. Therefore, the proposed 3D MRGB with GNDs is able to identify composite vibrational fingerprints in multiple biomolecules simultaneously for both TM and TE polarization. It also possesses the independent tunability for individual plasmonic resonance to satisfy different sensing applications.

**Figure 7:**
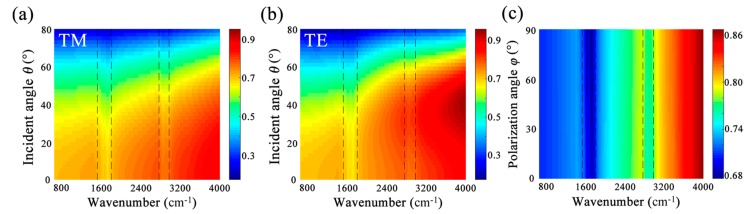
Transmittance of 3D MRGB covered with protein and lipid molecules as functions of wavenumber and incident angle *θ* of TM (**a**) and TE (**b**) polarization, as well as wavenumber and the polarization angle *φ* under normal incidence (**c**). Dashed vertical black strips outline the vibrational bands of protein and lipid molecules.

Next, we further investigate the sensing performance of 3D MRGB under non-normal mid-infrared incidence. In Figure 7a, transmittance spectra of 3D MRGB with protein and lipid as a function of wavenumber and incident angle *θ* are depicted for TM polarization. The parameters are *R*_1_ = 20 nm, *R*_2_ = 10 nm, *t*_1_ = 280 nm, *t*_2_ = 500 nm, *p* = 80 nm, *E_F_*_1_ = 0.6 eV, *E_F_*_2_ = 0.9 eV. When *θ* is below 72°, protein can be detected due to the spectral overlapping between the vibrational fingerprints and the first resonance. The vibrational fingerprint of lipid is also enhanced to some extent by the second resonance. When *θ* increases to 68°, the protein molecules can still be identified while the signal of lipid molecules is too weak to detect. In Figure 7b, for TE polarization, the vibrational fingerprints of protein is clearly detected until the incident angle is up to 70°. The lipid can be detected with a faint signal, and is almost undetectable when *θ* increases up to 65°. Therefore, the 3D MRGB can guarantee sensing ability in a wide incident angle range for both TM and TE polarized incidence.

Finally, the polarization dependence of 3D MRGB for detecting protein and lipid molecules is further evaluated. The transmission spectra under normal incidence with polarization angle *φ* from 0° to 90° are shown in Figure 7c. We assume the polarization angle of TM incidence to be 0°, so the transmittance in Figure 7c at *φ* = 0° is the same as the solid curves plotted in Figure 6b or Figure 6c. As *φ* increases to 90°, the incidence slightly becomes a TE polarized mid-infrared light. Therefore, the transmittance gradually turns out to be the same as the dashed curves in Figure 6b or Figure 6c, which is a little different from that of TM polarization. When 0° < *φ* < 90°, both *x*-axis and *y*-axis components coexist in the incident electric field, so the transmittance is shown as a non-polarized status. The vibrational fingerprints of protein are clearly observed at any polarization angle, while that of lipid is slightly weakened as the polarization angle increases. However, even if the polarization is up to 90° (TE polarization), a tiny vibrational signal located at the CH_2_ band can also be detected as shown in Figure 6b,c. When *E_F_*_1_ is fixed at 0.60 eV and *E_F_*_2_ is 0.90 eV, for TM polarization, the transmittance rates at 1532 cm^−1^ (amide-I), 1668 cm^−1^ (amide-II) and 2900 cm^−1^ (CH_2_) are 71%, 69% and 78%, respectively, while they are 72%, 70% and 77% for TE polarization. The small difference of sensing performance between TM and TE polarization is mainly because the unit cell of 3D MRGB is not four-fold symmetrical, although both the big GNDs and small GNDs can support plasmonic localized resonance in either *x*-axis or *y*-axis direction.

## 5. Conclusions

In summary, we have numerically designed a label-free MRGB with periodic GNRs of which each resonance band can be independently tuned by electrostatic gating. The decent detection performance for vibrational fingerprints in protein and lipid is revealed due to the tunable spectral selectivity and extreme field confinement of graphene. Electric field distributions are plotted to demonstrate the hotspots and investigate the physical mechanism. Geometric parameters are critical for the performance of MRGB and the robustness for oblique incidence is also elaborated. In addition, a MRGB with periodic GNDs is further developed to solve the issue of polarization dependence. We believe that the design principles in this paper will provide a significant guide in designing other multi-resonant SEIRA sensors based on graphene.
